# Killer Whale (*Orcinus orca*) Predation on Beaked Whales (*Mesoplodon* spp.) in the Bremer Sub-Basin, Western Australia

**DOI:** 10.1371/journal.pone.0166670

**Published:** 2016-12-06

**Authors:** Rebecca Wellard, Keith Lightbody, Leila Fouda, Michelle Blewitt, David Riggs, Christine Erbe

**Affiliations:** 1 Centre for Marine Science & Technology, Curtin University, GPO Box U1987, Perth, WA 6845, Australia; 2 Nature Photographer, Winthrop, WA 6150, Australia; 3 Marine Studies Institute, University of Sydney, Sydney, NSW 2006, Australia; 4 Riggs Australia Pty Ltd, PO Box 2076, Esperance, WA 6450, Australia; Maurice Lamontagne Institute, CANADA

## Abstract

Observations of killer whales (*Orcinus orca*) feeding on the remains of beaked whales have been previously documented; however, to date, there has been no published account of killer whales actively preying upon beaked whales. This article describes the first field observations of killer whales interacting with, hunting and preying upon beaked whales (*Mesoplodon* spp.) on four separate occasions during 2014, 2015 and 2016 in the Bremer Sub-Basin, off the south coast of Western Australia.

## Introduction

The killer whale (*Orcinus orca*) is a global species, occurring from shallow coastal waters to deep offshore waters [1]. They are the dominant oceanic apex predator, feeding on a variety of vertebrate and invertebrate species, including other marine mammals, seabirds, fish, sharks, squid and turtles [2]. Prey specialisations of killer whale communities in the Northern Hemisphere have been well documented over the last four decades and can be used to distinguish between sympatric non-interbreeding populations. For example, the eastern North Pacific Ocean comprises of three ecotypes: sympatric so-called “resident” and Bigg’s (formerly “transient”) communities, as well as so-called “offshore” killer whales [3–5]. Resident and offshore ecotypes feed on fish, which, in the case of resident killer whales, is predominantly Chinook salmon (*Oncorhynchus tshawytscha*) [3,6,7]; whereas Bigg’s killer whales consume almost exclusively marine mammals, with their diet including cetaceans and pinnipeds, and some seabirds and squid [3,8–10].

The North Atlantic comprises of three killer whale populations. Population A eats predominantly fish, mainly herring (*Clupea harengus*), however has been often observed switching between fish and marine mammals—indicative of a more generalist predator [11–14]. Population B lives sympatrically with Population A and contains two subpopulations: a generalist feeding on fish and mammals and a specialist feeding on mammals [12,15–17]. Whereas Population C has been reported to eat fish, including bluefin tuna (*Thunnus thynnus*) [11,14,15,18,19].

In Antarctic waters, killer whales have been designated into at least four ecotypes: Type A, Type B (pack ice killer whale), Type B (Gerlache killer whale), Type C and Type D [20,21]. These four ecotypes appear to have specialized diets. Types A and B pack ice killer whales have been reported to eat other cetaceans and pinnipeds [20–24], and the Gerlache killer whales have been noted to eat penguins [23]. Type C has been observed to eat fish; while the diet of Type D is virtually unknown, apart from the consumption of Patagonian toothfish (*Dissostichus eleginoides*) observed during longline interactions [18,20,23,24].

In Australian waters, there has been limited research on killer whales. Killer whales have been sporadically recorded in all state and territory waters. Higher concentrations have been reported off southern Australia, from southern New South Wales to western Victoria [25,26], and off Western Australia, from the far south-east to mid-north coast [27,28]. Seasonal trends in sightings in some locations may suggest fairly consistent occupancy that may coincide with aggregations of prey [25].

Despite killer whales being sighted in all Australian waters, most sighting data are incidentally collected during ecotourism encounters and from commercial fishers, with limited dedicated field research. Furthermore, there has been only one published account of feeding in Australian waters to date. Pitman et al. [28] observed killer whales off Western Australia preying on neonatal humpback whales (*Megaptera novaeangliae*) during the humpback whale northern migration to calving grounds. Beyond this, there have only been reports that killer whales in southern Western Australia also potentially feed on the Southern Ocean sunfish (*Mola ramsayi*) (DR personal observation, unpublished) and possibly an unidentified large squid (MB and DR personal observations, unpublished). This article describes, for the first time, field observations of killer whales preying upon beaked whales (*Mesoplodon* spp.) in the Bremer Sub-Basin, Western Australia, on four separate occasions during the months of February and March in 2014, 2015 and 2016.

## Methods

**Fig 1:**
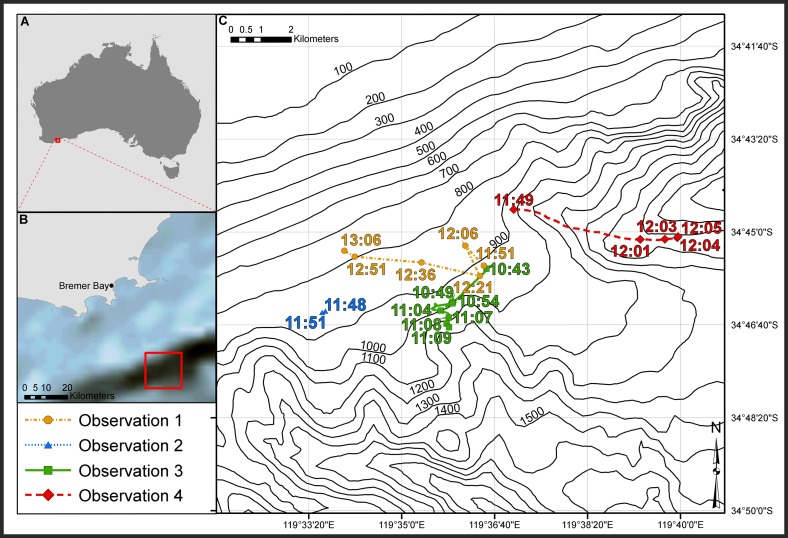
Study area offshore Bremer Bay where four separate predation events occurred. Map of Australia (A) shows the location of the canyon complex in the south-west. The box in (A) is expanded in (B) presenting this region in more detail. The box in (B) surrounds the locations where predations were observed and is expanded in (C). (Made with Natural Earth. Free vector and raster map data @ naturalearthdata.com).

The Bremer Sub-Basin is an area of approximately 11,500 km^2^, off the continental shelf of southwestern Australia, extending from Albany east towards Esperance. This sub-basin contains a complex system of submarine canyons with water depths ranging from 100 to 4500 m [29] (Fig 1). It is considered a biologically important area that attracts a myriad of species including fish, sharks, and deep-diving whales such as sperm whales (*Physeter macrocephalus*) [30]. The wider south-west marine region is further thought to be an important migratory area for humpback whales, and closer to shore, a calving area for Southern right whales (*Eubalaena australis*) [30].

Observations were made onboard commercial ecotourism vessels during daylight hours and variable weather conditions, during the months of January to April in 2014, 2015 and 2016. Whale-watching vessels departed from Bremer Bay, southern Western Australia and headed offshore, approximately 50 km south-east of Bremer Bay. Vessels, *Cetacean Explorer* and *The Southern Conquest*, were operated by Naturaliste Charters and Riggs Australia, respectively. A total of 141 field trips were conducted over the three field seasons in 2014, 2015 and 2016 throughout the months of January to April, with a killer whale sighting rate of 91.5%. All observations detailed here occurred in the months of February and March of these years.

For each encounter, we attempted to photograph all of the killer whales present using digital SLR cameras with telephoto zoom lenses, following established protocols for killer whale photo-identification studies [8,31,32]. Individual animals were identified by a combination of features, including any notches on the dorsal fin and fin shape, and differences in the eye patch and saddle patch pigmentation and shape.

Detailed behavioural observations of the predation events in 2014 and 2016 were made by RW. Opportunistic photographs and notes of predations in 2015 were collected by KL. Fig 1 illustrates the area where observations were made on four separate occasions in 2014, 2015 and 2016.

## Predatory Events

### Observation 1: 25 February 2014

**Fig 2:**
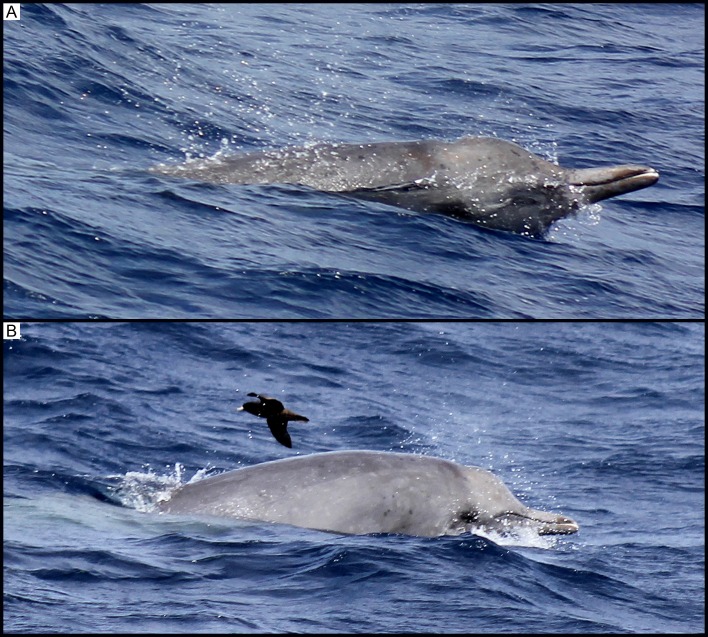
(A) and (B) Observation 1: A single mesoplodont beaked whale sighted in close proximity to the group of killer whales.

**Fig 3:**
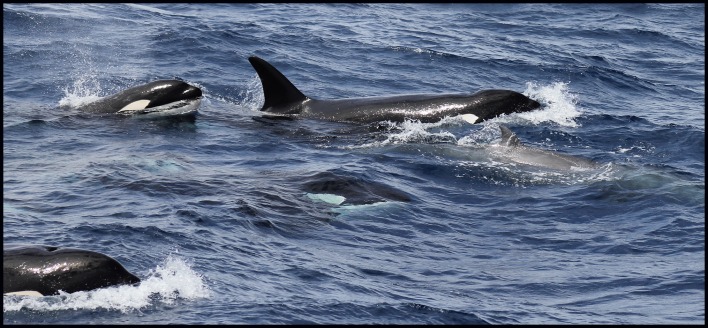
Observation 1: The beaked whale being flanked by killer whales closely on each side.

At 11:57, a group of a minimum of 20 killer whales was sighted off Bremer Bay approximately 36 km from shore (34°45’S, 119°36’E). Following initial observations, an unidentified cetacean was sighted within 1–2 m of the killer whales. After several surfacings, it was identified as a mesoplodont beaked whale (*Mesoplodon* spp., see Fig 2A and 2B). Over the next 67 minutes, the beaked whale was flanked by approximately five killer whales within 1 m on each side (Fig 3), with a further 10 to 15 killer whales dispersed within a 500 m radius of the group. Two other large adult male killer whales, identified by their tall dorsal fins, stayed approximately 800 m behind.

The killer whales that were surrounding the beaked whale were suspected to be adult females or sub-adult males/females, except for one juvenile. The killer whales continued to flank the beaked whale within 1 m, until at 12:32 the beaked whale broke off and headed towards our boat. The beaked whale was quickly intercepted by another killer whale that was previously with the larger, dispersed group. At 12:41 the beaked whale was resighted with several animals again flanking it, within 1–2 m on both sides. The killer whales continued to travel alongside the beaked whale at close proximity, and at 12:52, one killer whale appeared to be on top of the beaked whale just behind its dorsal fin, apparently submerging the beaked whale under the water. Upon the next surfacing, the killer whales were again flanking the beaked whale.

The first direct attack was observed at 13:00, one hour after the initial sighting, although it should be noted this may have occurred previously under the surface and not been observed. Two adult females and the juvenile charged at the flanks and the head of the beaked whale. Blood was visible on the beaked whale’s back and in the water. Upon the next surfacing, the beaked whale was surrounded by five killer whales, with the juvenile at the rear. The number of birds increased, particularly flesh-footed shearwaters (*Ardenna carneipes*), with many diving and feeding in the area.

At 13:01, the killer whales launched another attack, with two individuals striking the beaked whale’s left flank. It could not be confirmed whether the killer whales had their mouths open or closed (*i*.*e*., whether they were they biting or ramming), but blood was immediately visible in the water. During the next two surfacings, the beaked whale was visible from the rostrum to the dorsal fin, but no injuries were detected in the photos. It would appear any potential injuries were behind the dorsal fin or concealed under water. This was concurrent with the blood seen in the water towards the peduncle of the animal. Upon one of the beaked whale’s last surfacings, one killer whale came into contact with the animal’s left flank, but due to the position of the killer whale and an increase of water movement, it was unclear whether the killer whale’s mouth was open. It appeared that the killer whale was directly over the beaked whale’s dorsal fin and trying to submerge it. This occurred once again over the next three minutes, and other killer whales were observed underneath the beaked whale. During this time the amount of blood at the surface increased, along with the number of birds.

The beaked whale was last sighted at 13:04, with the juvenile killer whale at its head and two other killer whales on top of the beaked whale, pushing it below the surface. The killer whales were then observed conducting short dives around the vicinity of where the beaked whale was last seen. An increase in seabird foraging behaviour was noted at the water surface, with at least 70 flesh-footed shearwaters and two shy albatrosses *(Thalassarche cauta)* present. At 13:16, the killer whales engaged in surface-active social behaviour, including tail slapping. Bird foraging activity continued on the surface, and blood was evident in the water.

No flesh was observed in the mouths of the killer whales and although blood was seen in the water and on the beaked whale, it is possible that the killer whales had an unsuccessful attack on the beaked whale and it escaped, undetected by observers on the vessel. It is, however, probable that it was a successful kill, and the killer whales fed on the carcass below the surface undetected by the researchers. The two adult males remained distant throughout the attack, although this could be due to the fact they may not have been a part of the group. The killer whales started to disperse following their bout of social behaviour, and by 13:37 they had left the area.

### Observation 2: 8 February 2015

**Fig 4:**
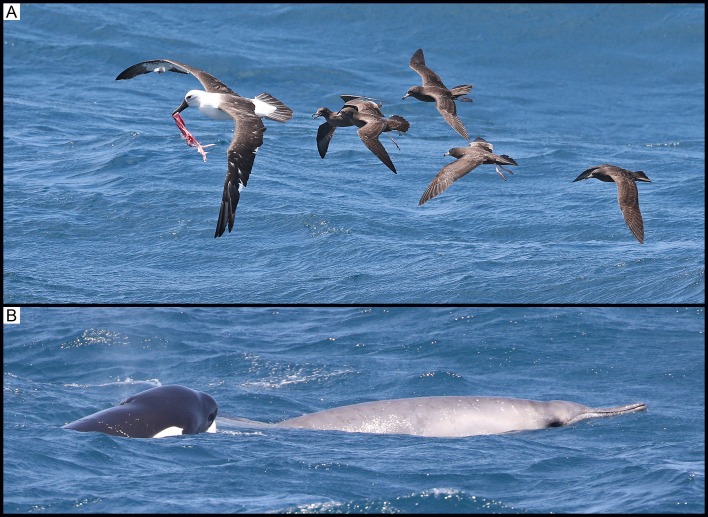
(A) Observation 2: Flesh observed in the beak of an Indian yellow-nosed albatross (*Thalassarche carteri*).(B) Observation 2: A single beaked whale surfacing in very close proximity next to a killer whale.

At 11:19, a group of at least seven killer whales, comprising of adult females, sub-adult males/females and juveniles, was sighted approximately 35 km from shore (34°46’S, 119°33’E). The animals showed an increase in surface activity. At 11:50, the birds in the area, namely Indian yellow-nosed albatross (*Thalassarche carteri*) and flesh-footed shearwaters, were photographed picking up red flesh from the surface of the water (Fig 4A). At 12:15, the killer whales increased their travelling speed and surged ahead. At 12:17, an unidentified beaked whale surfaced approximately 1 m from a killer whale (Fig 4B). This was the only time the beaked whale was seen surfacing. The beaked whale’s head and body forward of the dorsal fin were the only area visible at the surface. At 12:19, immediately after the sighting of the beaked whale on the surface, the killer whales surged through the water, with a high amount of surface activity. At 12:40, the killer whales exhibited social behaviour, including breaching and tail slapping. At 12:44, the birds were again noted on the surface diving and feeding.

Circumstantial evidence suggests that a predation event occurred, due to birds picking up flesh and a beaked whale immediately adjacent to the killer whales, though there was no photographic confirmation of flesh or bone inside the killer whale mouths.

### Observation 3: 17 February 2015

**Fig 5:**
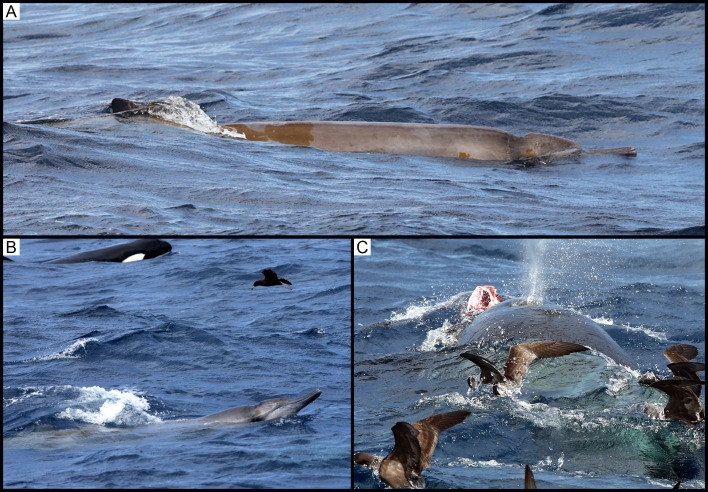
(A) Observation 3: A single beaked whale sighted in close proximity to the group of killer whales. (B) Observation 3: A single beaked whale sighted in close proximity to the group of killer whales. (C) Observation 3: A killer whale observed with flesh and bone in its mouth surrounded by birds foraging on the sea surface.

At 10:40, a group of at least six killer whales, comprising adult females, sub-adult males/females and juveniles, was sighted approximately 36 km from shore (34°45’S, 119°36’E). At 10:49, a mesoplodont beaked whale was sighted within 1 m of the killer whales (Fig 5A and 5B). The killer whales were both alongside and beneath the beaked whale, which was last seen at 10:55. At 11:04, bird activity increased in the immediate area; the species included flesh-footed shearwater, Indian yellow-nosed albatross, wandering albatross (*Diomedea exulans*) and white-faced storm-petrel (*Pelagodroma marina*). At 11:07, 12 minutes after the last sighting of the beaked whale, a killer whale was photographed with a carcass stripped of skin in its mouth (Fig 5C). At 11:08, the group of killer whales exhibited increased surface-active social behaviour, including breaching.

### Observation 4: 18 February 2016

**Fig 6:**
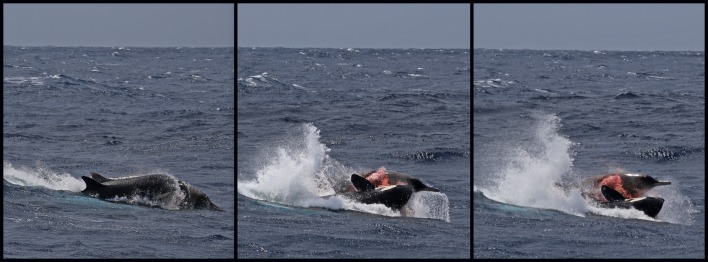
Observation 4: The beaked whale seen porpoising through the water with a killer whale attacking its right flank resulting in a large bite wound.

**Fig 7:**
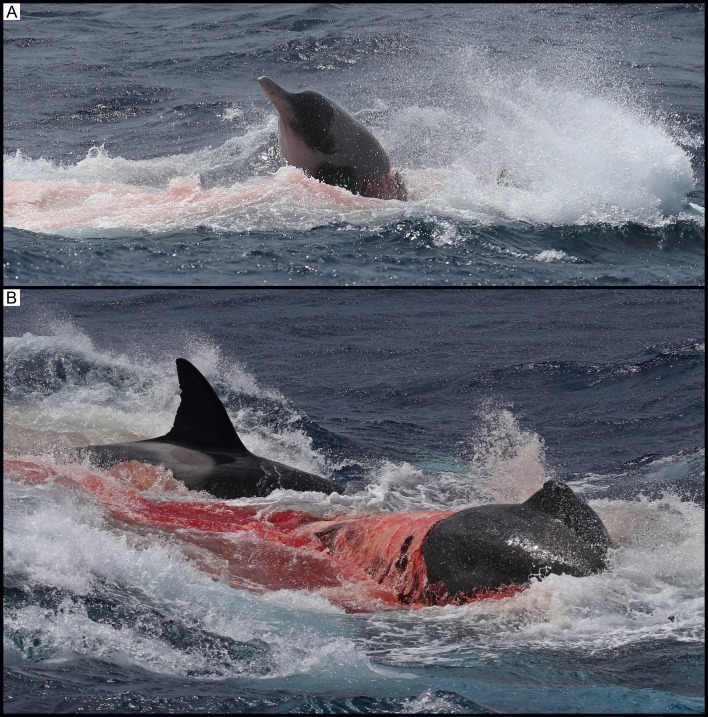
(A) Observation 4: The beaked whale’s head clear out of the water allowing for a positive identification of strap-toothed whale (*Mesoplodon layardii*). (B) Observation 4: The beaked whale with its skin stripped off the body from the rostrum to the dorsal fin.

**Fig 8:**
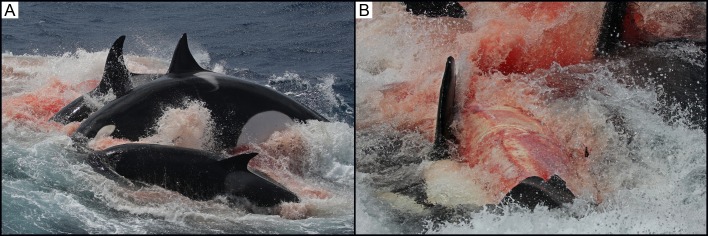
(A) Observation 4: Three killer whales synchronously attacking the beaked whale. (B) Observation 4: The beaked whale’s underside seen completely stripped of skin as the killer whales drag the beaked whale underwater.

At 11:10, a group of seven killer whales, comprising adult females, one adult male, sub-adult males/females and one calf, was sighted approximately 34 km from shore (34°43’S, 119°37’E). Animals were travelling in a south-west direction with consistent surfacing periods. At 11:43, the group suddenly changed direction and headed south-east at increasing speed. At 11:49, the group was traveling at a speed of approximately 18 knots. The first sighting of the beaked whale occurred at 12:03, roughly 5 km south-east (34°45’S, 119°39’E) from the original sighting. The beaked whale was seen porpoising through the water with one killer whale attacking its right flank, resulting in a large bite wound (Fig 6). There were repeated attacks on the beaked whale from both sides by at least four killer whales, including a calf, an adult female and male, and a sub-adult male/female. Additional killer whales had now joined this group, with at least three new killer whales sighted taking part in the attack. The beaked whale’s head was sometimes out of the water during these attacks allowing for a positive identification of strap-toothed whale based on the pigmentation pattern of the head (*M*. *layardii*; Fig 7A). The beaked whale was next seen at the surface, with considerable surface activity and splashing, and at least two killer whales alongside it. During this surface period, the killer whales stripped the skin off the body of the beaked whale from the rostrum to the dorsal fin (Fig 7B). At 12:04, three killer whales synchronously attacked the beaked whale (Fig 8A). Next, the beaked whale’s underside was seen completely stripped of skin and at least three killer whales dragged it underwater, leaving a large quantity of blood in the area (Fig 8B). This was the last sighting of the beaked whale. A hydrophone (High Tech Inc. HTI-96-MIN with built-in pre-amplifier; flat frequency response of 2 Hz to 30 kHz; sensitivity -164.1 dB re 1 V/μPa) was lowered over the side of the boat and vocalisations were recorded (SoundDevices 722 digital recorder sampling at 96 kHz, 24 bit).

Birds in the area, including Indian yellow-nosed albatross and flesh-footed shearwaters, were picking flesh from the surface of the water. The total number of killer whales observed in the area had increased to a minimum of 19 animals. Over the next 40 minutes, a large oil slick was seen on the surface and the killer whales slowly dispersed from the area.

## Discussion

Beaked whales (family Ziphiidae) are small to medium sized toothed whales (suborder Odontoceti), which are elusive and rarely sighted in Australian waters. The family Ziphiidae is one of the most wide-ranging families of cetaceans, however, knowledge about distribution and abundance of beaked whale species is limited [33]. What is known about beaked whale habitat preference indicates that they forage in deep water near the continental slope, in subsea canyons, or along steep-sided islands, and are often associated with cold-core eddy intrusions, which promote upwelling of nutrient-rich water [34–40]. In Australian waters, knowledge of their biology and distribution has been a result of intermittent sightings and stranding records [41–43].

The Western Australian coast has the highest species diversity (10) of beaked whale strandings compared to other Australian coasts, with a total of 74 Ziphiidae strandings from 1940 to 2010 [41]. Gray’s beaked whale (*Mesoplodon grayi*) was the most frequently reported stranded species (44%), and has been involved in the largest mass stranding (seven individuals) of all beaked whales stranded in Western Australia [41].

Most beaked whales are difficult to detect and identify at sea due to their elusive behaviour, low surfacing profile and preference for deep water. Detailed examination of the photographs from observations in 2014 and 2015 strongly suggests that these animals were long-beaked species of *Mesoplodon*; most likely Gray’s beaked whale and/or strap-toothed whale (*Mesoplodon layardii*) (R.Pitman, D.Coughran and C.Kemper, pers. comm.). Due to the absence of an erupted tooth and lack of adult colour patterning, these individuals were likely females or juveniles. The distribution of Gray’s and strap-toothed beaked whales supports this identification, with both species previously documented off Western Australia, and Gray’s beaked whale being the most commonly recorded species of beaked whales in Western Australian waters [41]. Photographs from Observation 4 in 2016 show distinct diagnostic features and allow for a positive identification of a strap-toothed whale (Fig 7A).

Killer whales feeding on beaked whale carcasses have been previously reported: a *Mesoplodon* sp. off Sri Lanka [44], a Cuvier’s beaked whale (*Ziphius cavirostris*) in the Mediterranean [45] and a northern bottlenose whale (*Hyperoodon ampullatus*) in Norway [46]. Jonsgård [46] also reported killer whales depredating harpooned beaked whales that were alive but tied to a vessel by harpoon rope. However, to our knowledge, there has not been a documented account of killer whales hunting and preying on beaked whales to date.

Additional evidence that killer whales prey on beaked whales is provided by playback experiments where pre-recorded killer whale calls were transmitted in close proximity to a tagged Blainville’s beaked whale (*M*. *densirostris*), which initiated avoidance behaviour at a very low received root-mean-square sound pressure level of 98 dB re 1 μPa, barely above the ambient noise level [47,48]. This beaked whale exhibited a prolonged avoidance response demonstrated by directed swimming over an extended period of time [47,48].

After a successful predation, killer whales are often observed exhibiting active social behaviour at the surface, such as pectoral fin and fluke slapping, breaching, and spyhopping [11,49,50]. Similar surface active behaviour was observed at the completion of the predatory events in the Bremer Sub-Basin. Social behaviour in killer whales is typically accompanied by vocalizations [51]. The calls detected during Observation 4 were mostly whistles of class BC01, BC02, BC04 and transition call BC09 [27]. These sounds were recorded during both social and travelling behaviour in 2014 and 2015, however, prior predations had not been observed, yet might have been missed.

Our observations indicated that only adult females, sub-adult males/females and juveniles were likely involved in the immediate predatory events of the beaked whales, while the adult males remained a distance behind. This behaviour is consistent with killer whale predations observed elsewhere [2,52,53]. Conversely, this is not exclusively the case, as adult males have been reported to take an active role in other predation events [44,54–56] and in 2016, we saw one adult male participate in the attack. While the role of adult males in predatory events seems to vary with prey type or specific feeding events, it appears that females and sub-adults take a stable role in marine mammal predations. Furthermore, juveniles are frequently seen to remain part of the hunting group, most probably in close association to their mothers [11,49,56].

Comparing identification photos across the years, there were no consistent groups sighted over the four predations. Amongst all observations, four individuals were resighted across multiple predations, either 2–3 times, with other individuals identified but only appearing once, although it is likely that not all animals were photographed during the events. The social and group dynamics of this population are still unknown and beyond the scope of this paper, highlighting the need for long-term population monitoring in this region.

Whilst the median group size for killer whales that were involved initially in each of the four beaked whale interactions in the Bremer Sub-Basin was 10 (range 6–20), a larger group of a further 10 or more killer whales either followed in close vicinity or appeared shortly after the kill. Interestingly, in Observation 4, killer whales that were not in the original sighting also joined in the attack of the beaked whale and the feeding. Mammal-eating killer whales have been noted for their small group size, whilst piscivorous killer whales and more generalist feeders have been observed to hunt in larger groups [3,11,16,18,57–59]. Baird and Dill [57] estimated that the optimal group size for North Pacific transients—mammal-hunting killer whales—is three individuals and suggested that larger groups of mammal-hunting killer whales would suffer a cost due to an increased probability of detection by prey. Other marine-mammal-eating killer whales also have been observed in small groups: two individuals for the Punta Norte, Patagonia population [58], and five for killer whales in Scottish waters [16]. However, it would appear small group size of mammal-eating killer whales is not cosmopolitan, with mammal-eating Type A and B Antarctic killer whales appearing in groups of 38 and 24 respectively [20,24], and 21 Type A killer whales attacking an Antarctic minke whale (*Balaenoptera bonaerensis*) [24] and eight killer whales in New Zealand attacking a pod of false killer whales (*Pseudorca crassidens*) [52].

There are other accounts of Bremer Sub-Basin killer whales potentially feeding on sunfish and an unidentified large species of squid (MB and DR personal observations, unpublished). Both are known prey species of killer whales [10,60–64]. Interestingly, specialized ecotypes, such as the North Pacific Bigg’s killer whales, which are known to feed primarily on marine mammals, have been shown to also prey on cephalopods [10]. It remains to be determined whether the killer whales observed feeding on squid and sunfish are the same population seen feeding on beaked whales, and therefore whether Bremer Sub-Basin killer whales have a broader prey base including non-mammalian prey. If there is an abundant and nutritious species of prey, then specialization may occur; while generalist feeding could be due to the lack of predictability and abundance of any specific prey type [28,65].

Very little is known about killer whales in Australian waters, their abundance, movements, behaviour, demographics, ecology or population status, and descriptions of their feeding behaviour and prey preferences is generally lacking. While encounters with killer whales are typically rare and unpredictable in Australian waters, the area offshore from Bremer Bay appears to support abundant killer whales during the austral summer, and provides an opportunity to study this little-known population.
